# Emotional Eating Patterns, Nutritional Status, and the Risk of Developing Type 2 Diabetes Among University Students: A Preliminary Assessment

**DOI:** 10.3390/healthcare13172186

**Published:** 2025-09-01

**Authors:** Víctor Manuel Jiménez-Cano, Adela Gómez-Luque, Vicente Robles-Alonso, María Valle Ramírez-Durán, Belinda Basilio-Fernández, Pilar Alfageme-García, Sonia Hidalgo-Ruiz, Juan Fabregat-Fernández, Alba Torres-Pérez

**Affiliations:** 1 Department of Nursing, University Centre of Plasencia, University of Extremadura, 10600 Plasencia, Spain; victormajc@unex.es (V.M.J.-C.); vroblesa@unex.es (V.R.-A.); bbasfer@unex.es (B.B.-F.); palfagemeg@unex.es (P.A.-G.); kirosony@unex.es (S.H.-R.); atorres@unex.es (A.T.-P.); 2 Department of Nursing, Faculty of Nursing and Occupational Therapy, University of Extremadura, 10004 Cáceres, Spain; juanfabregat@unex.es

**Keywords:** emotional eating, Type 2 diabetes risk, university students, FINDRISC, nutritional status, health promotion

## Abstract

**Background/Objectives:** Emotional eating has been linked to neurobiological mechanisms similar to those observed in addictive behaviors, and this occurrence appears not fully understood, even in academic communities/environments. To supplement existing information, a preliminary assessment of university students’ emotional eating patterns, nutritional status, and the risk of developing type 2 diabetes has been performed in this current work. **Methods:** A cross-sectional study was conducted with 129 students from the University of Extremadura, Spain. Emotional eating was assessed using the Emotional Eating Questionnaire (EEQ) and the Three-Factor Eating Questionnaire—Spanish version (TFEQ-R18(SP)) questionnaires, while type 2 diabetes risk was evaluated through the Finnish Diabetes Risk (FINDRISC) score test. Anthropometric data, including height, weight, BMI, and body composition, were collected using standardized measurement protocols. Statistical analyses included ANOVA and Kruskal–Wallis tests. **Results:** Significant differences were observed in BMI (mean ± SD: 23.93 ± 5.36 kg/m^2^) and total EEQ score (mean ± SD: 9.82 ± 5.82) across FINDRISC risk categories (*p* < 0.001 and *p* = 0.001, respectively). The Very Emotional Eaters group presented higher BMIs and FINDRISC scores, along with greater score dispersion. No significant differences were identified in EEQ subscales. **Conclusions:** Emotional eating behaviors may serve as indirect indicators of metabolic vulnerability, particularly when combined with excess body weight. These findings underscore the importance of incorporating emotional regulation and mindful eating strategies into health promotion programs for young adults.

## 1. Introduction

Uncontrolled eating behavior is influenced by multiple individual, interpersonal, and environmental factors and has been linked to neurobiological alterations similar to those observed in other addictions, such as activation of the dopaminergic reward system [1]. Obesity, the prevalence of which continues to rise, has emerged as a global epidemic and a major public health concern. According to recent data from the World Obesity Federation, more than 878 million adults were living with obesity in 2022 [2], and this number is projected to exceed 1.1 billion by 2030, affecting nearly half of the world’s adult population [2,3]. These trends highlight an alarming and accelerated increase in recent years, underscoring the urgent need for effective prevention and treatment strategies. Food addiction is strongly associated with excessive caloric intake, which contributes to adverse health outcomes such as elevated Body Mass Index (BMI), Type 2 Diabetes Mellitus (T2DM), and cardiovascular disease [4,5]. These effects are further exacerbated by insufficient physical activity, creating two interrelated behavioral risk factors that act synergistically to promote chronic disease development [6]. Moreover, these factors are closely linked to outcomes measured by the Finnish Diabetes Risk (FINDRISC) score, a validated tool used to estimate the risk of developing T2DM in adults [7]. The FINDRISC questionnaire includes items assessing physical activity habits (e.g., engaging in at least 30 min of daily exercise) and dietary behaviors, particularly the regular consumption of fruits and vegetables. Although this tool does not specifically evaluate food addiction, the condition is characterized by unbalanced eating patterns, including high consumption of ultra-processed foods and low fiber intake [5]. Such behaviors may indirectly contribute to unfavorable scores in the dietary dimension of the test [8].

University students in developed countries often exhibit dietary patterns marked by high consumption of ultra-processed foods, sugary beverages, and snacks rich in saturated fats and refined carbohydrates. These habits are influenced by academic stress, irregular schedules, and limited nutritional awareness, particularly in the European Union, where young adults frequently report low adherence to healthy dietary models such as the Mediterranean Diet [9]. In Spain, studies show that university students tend to skip meals, rely on convenience foods, and consume excessive amounts of sweets and soft drinks, leading to poor diet quality and an increased intake of saturated fats and alcohol [10,11]. These behaviors are concerning given the rising prevalence of type 2 diabetes across Europe, where over 61 million adults are affected [12]. Spain ranks as the second country in the EU with the highest diabetes prevalence, affecting 14.8% of the adult population—approximately 5.1 million people [13]. Emotional eating, defined as the consumption of food in response to negative emotions such as stress or anxiety, typically involves palatable, energy-dense foods and functions as a maladaptive emotion regulation strategy [14,15]. This behavior has been associated with an increased risk of obesity, metabolic syndrome, and T2DM, as demonstrated in several recent studies [16,17,18,19]. In contrast, food addiction denotes addiction-like patterns—such as craving, loss of control, tolerance, and persistent consumption despite adverse consequences—more closely linked to ultra-processed foods and reward system dysregulation [4,5]. Although conceptually related, these constructs are distinct; accordingly, the present study focuses on emotional eating as operationalized with the EEQ and the emotional eating domain of the TFEQ-R18(SP), rather than on food addiction [14,15]. Emotional eating has been linked to increased caloric intake, poor nutritional choices, and a higher BMI, all of which contribute to metabolic dysregulation and elevated risk of type 2 diabetes [18,19,20]. Emotional eating is especially prevalent among university students, who face unique psychosocial stressors and transitional life stages that may trigger maladaptive coping mechanisms [10,11,12,13,18]. Understanding the connection between emotional eating and metabolic risk is essential, as these behaviors not only influence weight gain but also exacerbate insulin resistance and other components of metabolic syndrome [19].

Eating behaviors are influenced by multidimensional internalized traits, encompassing behavioral, cognitive, and affective components [21]. To assess these dimensions, several validated instruments have been developed, including the Emotional Eating Questionnaire (EEQ) [15], which identifies emotional eating patterns such as preference for high-calorie foods and loss of control, and the Three-Factor Eating Questionnaire (TFEQ-R18 (SP)) [9], which evaluates cognitive restraint, emotional eating, and uncontrolled eating. These behavioral traits are associated with Body Mass Index (BMI) and diabetes risk, as reflected in FINDRISC scores [14,16]. Both questionnaires have demonstrated adequate reliability and validity in Spanish-speaking populations, particularly in studies examining eating behavior and BMI [14,15], and are considered practical tools for evaluating emotional regulation and dietary habits, supporting their use in promoting healthy lifestyle behaviors. Given their psychometric robustness and relevance to the study population, exploring the relationship between these measures and BMI may provide valuable insights into how emotional factors and diet quality interact to influence body weight and diabetes risk [14,21,22].

To supplement existing information, therefore, a preliminary assessment of university students’ emotional eating patterns, nutritional status, and risk of developing T2DM is conducted in the present study. The idea here is to examine whether emotional eating patterns—assessed using the EEQ and TFEQ-R18 (SP) questionnaires—are associated with nutritional status and the risk of developing T2DM, as estimated by the FINDRISC test. This study seeks to determine whether greater emotional dysregulation in eating behavior correlates with higher BMI and less favorable metabolic risk indicators. Understanding these associations in young university populations could offer critical insights for developing preventive strategies within both academic and clinical settings.

## 2. Materials and Methods

### 2.1. Research Design

This study employed a descriptive, cross-sectional observational design, following the STROBE (Strengthening the Reporting of Observational Studies in Epidemiology) guidelines [23] to ensure methodological rigor and transparency. The research was conducted within the framework of a broader project titled “Risk of Type 2 Diabetes in University Students at the University of Extremadura: A Cross-Sectional Study” [24], which itself was based on the protocol of a randomized trial evaluating an online educational intervention for prediabetic individuals aged 18–45 years [25].

The FINDRISC questionnaire was used as a validated screening tool to estimate diabetes risk [14], while eating behaviors were assessed using two psychometrically robust instruments: the TFEQ-R18(S-P) [14,26] and the EEQ [15,27]. To minimize interprofessional bias and ensure consistency in data collection, three nurses from the research team were trained according to a standardized protocol for anthropometric measurements and questionnaire administration, following nutritional assessment procedures described in previous studies [14]. Their selection was based on availability and prior experience in fieldwork, and all received specific training to ensure data reliability. All questionnaires were self-administered online through a secure platform, and technical support was offered via email and WhatsApp in case participants required guidance.

### 2.2. Participants

**Figure 1:**
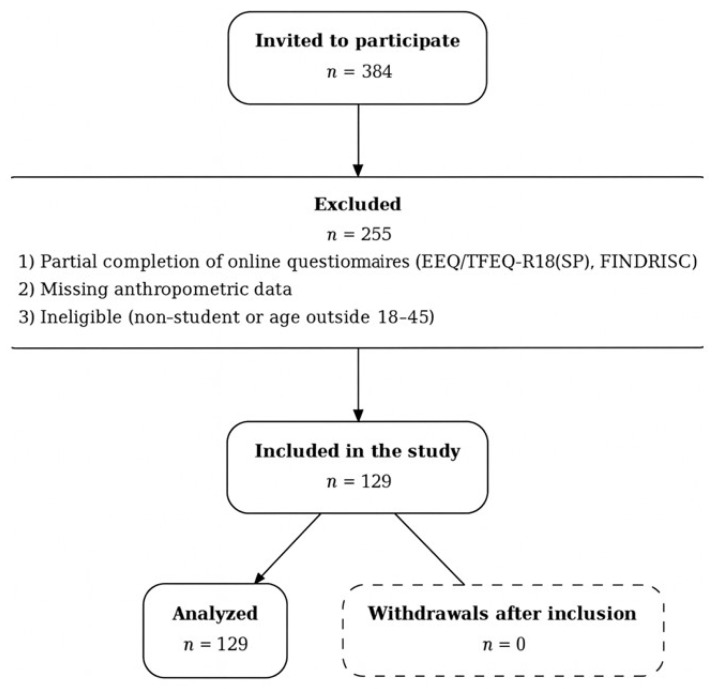
Flowchart of the participant recruitment process.

The recruitment process is summarized in Figure 1. The minimum required sample size was calculated a priori with G*Power 3.1 for a one-way ANOVA (medium effect size f = 0.25, α = 0.05, ** power = 0.80), yielding *n* = 128 [28]. To mitigate attrition and incomplete responses, 384 students were invited. Of these, 255 were excluded primarily due to partial completion of the online questionnaires (e.g., missing items in EEQ/TFEQ-R18(SP) or FINDRISC) and missing anthropometric data; a smaller subset did not meet eligibility criteria after screening (non-student status or age outside 18–45). The final sample comprised 129 participants (83 females (64.3%), 46 males (35.7%); mean age = 22.71 ± 5.22 years). Recruitment was conducted at the University of Extremadura (Cáceres, Spain) between November 2022 and March 2023 using convenience sampling. Participants were contacted via institutional email and provided electronic informed consent; additional recruitment strategies included QR-coded posters, social media outreach, classroom visits, and follow-up calls. Eligibility criteria are detailed below in Section 2.2.1 and Section 2.2.2.

#### 2.2.1. Inclusion Criteria

Participants had to meet two eligibility conditions: (1) age between 18 and 45 years; and (2) current enrollment at the University of Extremadura. Age eligibility was verified using the date of birth reported in the online screening form, and enrollment status was confirmed against the institutional student registry.

#### 2.2.2. Exclusion Criteria

Participants were excluded if they reported: (1) a previous medical diagnosis of diabetes; (2) physical limitations incompatible with moderate physical activity (i.e., inability to engage in approximately 30 min/day of moderate-intensity activity); or (3) non-student status at screening. These conditions were assessed through the online screening questionnaire and verified, when applicable, against recruitment records (e.g., student registry). Exclusion criteria were applied prior to data collection and are summarized in the recruitment flowchart (Figure 1).

The study protocol was registered (OSF: 10.17605/OSF.IO/4D8FJ) and developed in accordance with the SPI-RIT 2013 guidelines [29]. Ethical approval was granted by the Bioethics Committee of the University of Extremadura (No. 165//2021). All procedures complied with the Declaration of Helsinki [30] and national biomedical research regulations. Confidentiality was ensured by anonymizing data with alphanumeric codes. Only principal investigators accessed the final dataset, which will be securely stored for five years. No participant requested withdrawal.

### 2.3. Measuring Instrument and Procedure

Participants completed the TFEQ-R18(S-P) [14] and the EEQ [15]. The TFEQ-R18(S-P) is a validated instrument that assesses three dimensions of eating behavior: cognitive restraint, uncontrolled eating, and emotional eating. It contains 18 items rated on a 4-point Likert scale and has demonstrated good internal consistency, with Cronbach’s alpha coefficients ranging from 0.75 to 0.87 [31].

The EEQ includes 10 items rated on a 4-point Likert scale (0 = never to 3 = always), measuring emotional eating through three subscales: lack of control, preference for high-calorie foods, and guilt. It has demonstrated adequate reliability (α = 0.61–0.77) and test–retest stability (*r* = 0.702; *p* < 0.001) [15]. The FINDRISC questionnaire, validated in multiple European populations [14], was used to estimate the 10-year risk of developing T2DM. For analysis, participants were grouped into three risk categories based on total FINDRISC scores: low risk (<7), moderate risk (7–11), and high risk (≥12), following prior recommendations [8]. Anthropometric measurements were taken using a Seca 213 stadiometer (height) and a Tanita MC 780 MA bioimpedance analyzer (weight and body composition). Nutritional status was classified according to the World Health Organization’s BMI cut-off points: underweight (<18.5 kg/m^2^), normal weight (18.5–24.9 kg/m^2^), overweight (25.0–29.9 kg/m^2^), and obesity (≥30.0 kg/m^2^) [32].

### 2.4. Statistical Analysis

After checking the normality of the data (Kolmogorov–Smirnov test, *p* > 0.05). The data do not follow a normal distribution, *p* < 0.05. BMI *p* < 0.001; Findrisk test *p* < 0.001; EEQ *p* < 0.001; cognitive restraint *p* < 0.001; emotional eating *p* < 0.001; uncontrolled eating *p* = 0.001. A descriptive analysis was applied, and the nonparametric Kruskal–Wallis test, ranges of means, was used to determine the BMI and the differences according to the EEQ. All statistical calculations were performed using SPSS version 25.0 (IBM, Armonk, NY, USA) (license UEX campus), establishing a significance level of 5% (*p* < 0.005).

## 3. Results

### 3.1. Baseline Characteristics of the Participants

The sample consisted of 129 university students from the University of Extremadura, with a mean age of 22.71 years (SD = 5.22), ranging from (minimum age) to (maximum age) years. Of the total participants, 64.3% (*n* = 83) were women and 35.7% (*n* = 46) were men. Regarding Body Mass Index (BMI) distribution, 60.47% of participants were classified within the normal weight range, while 24.03% were overweight. Underweight cases accounted for 7.75%, and obesity was observed in 5.43% for Class I, 0.78% for Class II, and 1.55% for Class III. In terms of the risk of developing T2DM, as measured by the FINDRISC questionnaire, the majority (85.27%) fell into the low-risk category, followed by 8.53% in the moderate-risk range and 6.20% with slightly elevated risk. Regarding eating behavior, assessed through the EEQ, the largest group was Low Emotional Eaters (50.4%), followed by Emotional Eaters (24.0%), Non-Emotional Eaters (17.8%), and Very Emotional Eaters (7.8%).

This initial characterization provides essential clinical and behavioral context for analyzing associations between anthropometrically assessed nutritional status, T2DM risk (FINDRISC), and emotional eating among university students.

### 3.2. Descriptive Statistics and Comparative Analysis

Six primary variables related to metabolic health and eating behavior patterns were analyzed: BMI, FINDRISC score, overall score on the EEQ, and the latter’s three subscales—cognitive restraint, emotional eating, and uncontrolled eating.

The mean values for the sample were as follows: BMI = 23.93 (SD = 5.355), FINDRISC = 4.26 (SD = 3.472), total EEQ score = 9.82 (SD = 5.817), cognitive restraint = 14.34 (SD = 2.884), emotional eating = 9.29 (SD = 1.969), and uncontrolled eating = 25.50 (SD = 4.144). These indicators reflect a predominantly normal-weight population with intermediate levels of emotional eating behaviors. A detailed summary of the means, standard deviations, and Kruskal–Wallis test results for each variable is presented in Table 1. To assess whether these variables differed significantly across levels of diabetes risk as measured by the FINDRISC test, a non-parametric Kruskal–Wallis test was applied. Results revealed statistically significant differences in BMI (H = 24.29; *p* < 0.001) and the overall EEQ score (H = 23.58; *p* = 0.001). These findings suggest a positive relationship between diabetes risk, increased body weight, and the presence of emotional eating behaviors, underscoring the potential clinical relevance of the EEQ as a complementary tool for identifying profiles of metabolic vulnerability in university students. Conversely, no significant differences were observed across T2DM risk categories (FINDRISC) for the EEQ subscales—cognitive restraint (H = 3.933, *p* = 0.686), emotional eating (H = 2.876, *p* = 0.824), and uncontrolled eating (H = 8.557, *p* = 0.200)—indicating that, in this sample, these subdomains did not vary by current diabetes-risk category.

### 3.3. Distribution of Diabetes Risk Scores (FINDRISC)

**Figure 2:**
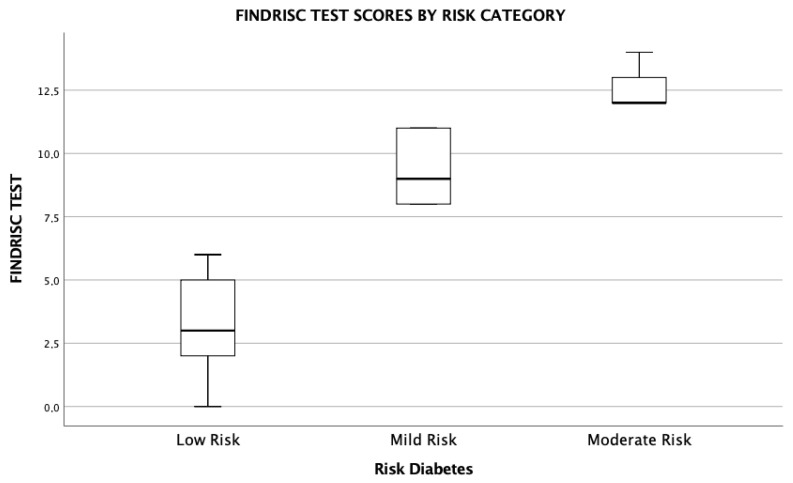
Distribution of FINDRISC Test Scores by Diabetes Risk Category.

Figure 2 illustrates the graphical distribution of FINDRISC scores, grouped into three diabetes risk categories: low, moderate, and medium. The diagram reveals an upward trend: as the risk category increases, so does the median score, indicating that participants classified at higher risk tend to present higher values on the questionnaire. Additionally, greater score dispersion is observed within the moderate and medium risk groups, suggesting higher individual variability in these profiles.

These findings support the sensitivity of the FINDRISC test in differentiating among varying levels of diabetes risk in a university student population.

### 3.4. Emotional Eating Profiles and Their Relationship with BMI and Diabetes Risk

To examine how emotional eating patterns relate to anthropometrically assessed nutritional status and T2DM (FINDRISC), participants were classified into four profiles according to their EEQ scores: Low Emotional Eaters, Emotional Eaters, Non-Emotional Eaters, and Very Emotional Eaters. The distribution of these profiles within the sample showed a predominance of Low Emotional Eaters (50.4%), followed by Emotional Eaters (24.0%), Non-Emotional Eaters (17.8%), and Very Emotional Eaters (7.8%). This segmentation allowed for a comparative analysis of BMI and FINDRISC scores as indicators of T2DM.

**Figure 3:**
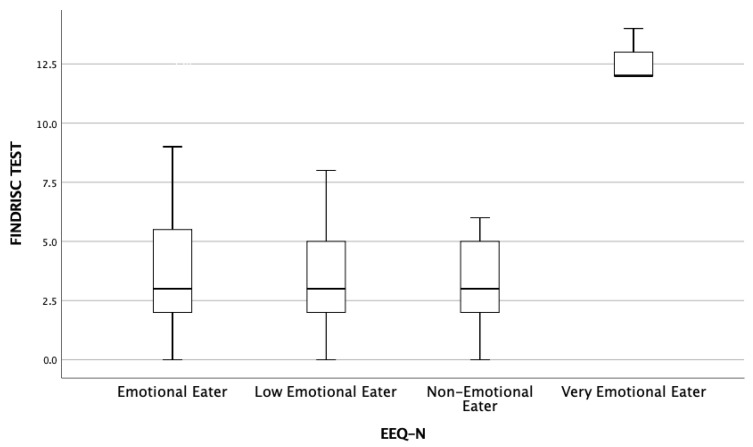
Distribution of FINDRISC scores by emotional eating profile.

Descriptive analyses revealed that participants classified as Very Emotional Eaters had higher median FINDRISC scores, accompanied by greater score dispersion. Similarly, this group also exhibited slightly higher BMI values compared to the other profiles. Although no formal inferential tests were conducted in this section and no statistically significant differences were identified, these observed trends are clinically relevant. Figure 3 illustrates the distribution of FINDRISC scores across the four emotional eating profiles. A progressive increase in the median score is evident as the level of emotionality in eating behavior rises, with the Very Emotional Eaters group showing the highest median and the widest variability. These findings suggest that greater emotional reactivity to food intake may be associated with a more vulnerable metabolic profile. This evidence reinforces the importance of incorporating behavioral indicators, such as emotional eating, into preventive strategies for metabolic disorders, particularly within university student populations.

### 3.5. Summary of Key Findings

The findings of this study reveal significant patterns between anthropometric indicators of nutritional status, emotional eating behavior, and type 2 diabetes risk among university students. Statistically significant differences were observed in BMI and the overall EEQ score across different risk levels as assessed by the FINDRISC test, suggesting that body weight and emotional eating behaviors are linked to metabolic vulnerability. No significant differences were found in the specific EEQ subdimensions (cognitive restraint, emotional eating, and uncontrolled eating), indicating that these traits may remain relatively stable regardless of T2DM risk level.

Additionally, emotional eating profiles—particularly the Very Emotional Eaters group—displayed higher median scores and greater variability in both BMI and FINDRISC scores. This pattern visually reinforces the potential connection between heightened emotionality in eating behavior and increased type 2 diabetes risk.

## 4. Discussion

The results partially confirm the proposed hypothesis, revealing an association between BMI and T2DM risk, as well as a positive trend between emotional eating and higher FINDRISC scores. These findings suggest that both nutritional status and emotionally driven eating behaviors may play an important role in the early detection of metabolic vulnerability among university students.

From a public health perspective, emotional eating among university students is a modifiable behavioral correlate of early T2DM risk. University settings are suitable for case-finding with simple risk tools (e.g., FINDRISC [8]) and for delivering brief, evidence-based education through student health services. Evidence shows that interventions including emotion regulation and mindful eating can improve diet-related behaviors and metabolic indicators in adults at risk or with DM2 [33,34], and lifestyle programs have delayed diabetes in Spanish primary care [27]. Given the prevalence of emotional eating reported in university samples [34] and the associations observed here, incorporating these components into routine student health promotion may help reduce early T2DM. These actions should be aligned with obesity-prevention and physical-activity initiatives [6] and evaluated using objective metabolic measures in future studies.

Consistent with previous studies, the mean BMI observed in this sample (23.93 ± 5.355) places most participants within the normal-weight range, although a non-negligible prevalence of overweight and obesity was identified. These results are comparable to those reported by González [35], who documented a mean BMI of 24.80 kg/m^2^ with a similar weight distribution: underweight (1.22%), normal weight (54.88%), overweight (36.59%), and obesity (7.325%). Similarly, Bernabéu et al. [15] provided psychometric evidence regarding EEQ patterns among Spanish students, while Sosa-Cordobés et al. [34] found that 25.85% of university students were overweight and 66.6% exhibited emotional eating, reinforcing the clinical relevance of BMI as an early indicator.

Regarding emotional eating profiles, 50.4% of participants in this study were classified as emotional eaters, a proportion consistent with findings from other university samples. Although the EEQ subscales (cognitive restraint, emotional eating, and uncontrolled eating) did not show significant differences across FINDRISC risk categories, higher scores—particularly in terms of variability—were observed among the Very Emotional Eaters group. This trend suggests that a greater emotional component in eating behavior may correlate with less favorable metabolic profiles, even if this association was not confirmed at an inferential level.

Our findings are consistent with prior research linking emotional eating to metabolic risk indicators. Mustač et al. [19] identified significant associations between psychological eating patterns and both T2DM and metabolic syndrome, reinforcing the behavioral dimension of early metabolic vulnerability. Ramírez-Garza et al. [18] further demonstrated that emotional eating and poor diet quality were associated with oxidative stress and adiposity in adolescents, suggesting that these behaviors may contribute to subclinical metabolic alterations even in young populations. Chew et al. [16], in a meta-analysis, confirmed that weight loss interventions targeting emotional eating led to improvements in BMI and glycemic control. These comparisons support the notion that emotional eating, particularly when assessed globally rather than by subdomains, may serve as a practical behavioral marker for early screening.

While emotional eating has been widely studied in university populations [15,34], its direct association with validated metabolic risk tools—such as FINDRISC—has received limited attention. Kes [36] is among the few who applied FINDRISC in this context, although emotional eating did not show significant predictive value in their sample. Our study extends this literature by demonstrating that overall EEQ scores are significantly associated with BMI and diabetes risk categories, even in a young, non-clinical population. By integrating validated psychometric tools with FINDRISC, our study contributes to a growing body of evidence advocating for multidimensional approaches to diabetes prevention in young adults.

These observations align with Kes [36], who also reported no statistically significant association between T2DM risk and emotional eating profiles, despite identifying sex-based differences in FINDRISC classification. However, other studies present contrasting evidence: Carrière et al. [37] and Miller et al. [33] demonstrated that interventions focused on mindful eating and emotional regulation can improve dietary habits and metabolic indicators, including glycemic control in adults at high risk or with a confirmed diagnosis of T2DM. These findings support the potential utility of incorporating such components into university-based health promotion programs.

The elevated prevalence of emotional eating in our sample may reflect the unique psychosocial pressures faced by university students, including academic stress, irregular schedules, and increased autonomy in food choices [34,38]. These factors may contribute to emotionally driven eating behaviors that, over time, influence metabolic risk. Interestingly, while the global EEQ score was associated with BMI and FINDRISC categories, the subscales did not show significant differences. This may suggest that emotional eating operates as a multidimensional construct, and that aggregated scores better capture its impact on health outcomes. Future studies should explore whether specific emotional triggers or coping styles mediate this relationship. By situating our findings within this behavioral and contextual framework, we aim to deepen understanding of how and why emotional eating contributes to early metabolic vulnerability in young adults. While these findings offer valuable insights, several methodological and contextual limitations must be considered to properly interpret their implications.

Study limitations should be acknowledged to contextualize the present findings. First, the relatively small sample size may have reduced statistical power and limited the generalizability of results, particularly in subgroup analyses such as the “Very Emotional Eaters” profile. This constraint positions the current work as a preliminary investigation. Second, the exclusive use of self-administered instruments (FINDRISC, EEQ, TFEQ-R18(SP)) introduces potential biases, including recall inaccuracies and social desirability effects, which may have influenced the observed associations. Third, the cross-sectional design precludes causal inference, restricting the ability to determine temporal relationships among emotional eating, BMI, and metabolic risk. Furthermore, the absence of objective clinical indicators (e.g., fasting plasma glucose, lipid profile, waist circumference) limits the precision of risk stratification. Challenges encountered during data collection—such as limited participant availability and variability in response accuracy—may have further impacted data quality and subgroup comparisons. Future research should address these limitations by employing longitudinal designs, incorporating objective metabolic measures, and recruiting larger, more diverse university samples to enhance external validity.

Future research should involve larger and more diverse university samples and adopt longitudinal designs. In addition, mixed-methods approaches that integrate quantitative outcomes (e.g., EEQ/TFEQ-R18(SP), FINDRISC, BMI) with qualitative interviews or focus groups can clarify triggers, context, and mechanisms linking emotional eating to early diabetes risk. Experimental designs—particularly randomized controlled trials conducted within student health services—should test brief, theory-based emotion regulation and mindful-eating components, assessing changes in eating behavior alongside objective metabolic outcomes (e.g., fasting plasma glucose, HbA1c, lipid profile, waist circumference, and blood pressure) with follow-up beyond one semester. Including psychosocial variables (academic stress, sleep quality, physical activity, personality traits) as potential moderators or mediators is recommended. Existing evidence indicates that such programs can improve diet-related behaviors and metabolic indicators [33,37] and that lifestyle interventions have delayed diabetes in Spanish primary care [27]; integrating physical-activity targets may further enhance outcomes [6,21].

## 5. Conclusions

In this university cohort, elevated scores on the Emotional Eating Questionnaire (EEQ) were associated with higher BMI and increased metabolic risk, as measured by the FINDRISC tool. Although EEQ subscale patterns did not consistently differentiate risk levels, individuals classified as Very Emotional Eaters showed greater variability in both BMI and FINDRISC scores, suggesting clinical relevance for this subgroup. These findings support the use of emotional eating as a behavioral marker for the early identification of students at risk for metabolic disorders. Given that nutritional status was assessed through anthropometric measures, emotional eating may serve as a practical proxy for underlying metabolic vulnerability.

From a preventive health perspective, university health services could implement brief risk screening (e.g., FINDRISC), routinely assess emotional eating behaviors, and offer targeted interventions focused on emotion regulation, mindful eating, and lifestyle counseling. Future research should validate these findings in larger, longitudinal cohorts using objective metabolic indicators (e.g., fasting glucose, HbA1c, lipid profile, waist circumference, and blood pressure), and explore mediating psychosocial factors such as academic stress, sleep quality, and physical activity.

## Figures and Tables

**Table 1:** Descriptive statistics and Kruskal–Wallis test results by diabetes risk level (fINDRISC).

Variables	Means	SD	H Kruskal–Wallis	Df ^1^	*p*-Level
IMC	23.93	5.355	24.29	6	<0.001
Test Findrisc	4.26	3.472	-	-	-
EEQ	9.82	5.817	23.58	6	0.001
Cognitive Restriction	14.34	2.884	3.933	6	0.686
Emotional Eating	9.29	1.969	2.876	6	0.824
Uncontrolled Eating	25.50	4.144	8.557	6	0.200

^1^ df: degrees of freedom.

## Data Availability

The data presented in this study are not publicly available due to privacy restrictions involving individual health and behavioral records of participants.

## Abbreviations

The following abbreviations are used in this manuscript:

BMI	Body Mass Index
T2DM	Type 2 Diabetes Mellitus
EEQ	Emotional Eater Questionnaire
TFEQ-R18(SP)	Three-Factor Eating Questionnaire—Spanish version
FINDRISC	Finnish Diabetes Risk Score
UEX	University of Extremadura
STROBE	Strengthening the Reporting of Observational Studies in Epidemiology
SPSS	Statistical Package for the social sciences
